#  Effects *of Siraitia grosvenorii* Fruits Extracts on Physical Fatigue in Mice 

**Published:** 2013

**Authors:** Da-Duo Liu, Xue-Wu Ji, Rong-Wei Li

**Affiliations:** a *Physical Education Institute of Jilin Normal University, Siping City, Jilin 136000, China.*; b *Department of Physical Education of Hebei Normal University of Science and Technology, Qinhuangdao City, Hebei 066004, China. *

**Keywords:** *Siraitia grosvenorii* fruits extracts, Physical fatigue, Mice, Forced swimming test

## Abstract

In this study, the effects of *Siraitia grosvenorii *fruits extracts (SGFE) on physical fatigue were investigated. One hundred and forty-four mice were randomly divided into four groups: control group, low-dose SGE-treated group, middle-dose SGE-treated group and high-dose SGFE-treated group. The animals of control group received an oral administration of physiological saline in a volume of 2.5 mL, and the animals of treated group received the same volume of SGFE (100, 200 and 400 mg/Kg bodyweight, once a day) for 28 days. After 28 days, anti-fatigue effects of SGFE were assessed 10 h after the last treatment by forced swimming test and some biochemical parameters related to fatigue, including blood lactic acid, serum urea nitrogen, liver glycogen and muscle glycogen were measured. The data showed that SGFE can extend the swimming time of the mice, as well as increasing the liver and muscle glycogen contents, but decrease the blood lactic acid and serum urea nitrogen levels. These results indicated that *Siraitia grosvenorii *fruits extracts had significant anti-fatigue effects on mice and these effects were dose-dependent.

## Introduction

Fatigue is a common symptom both in sickness and in health and it is best defined as difficulty in initiating or sustaining voluntary activities (1, 2). It can be subdivided into physical and mental fatigues. For physical fatigue, it is generally accepted that muscle fatigue mainly arises in muscle tissue from a disorder of energy metabolism, not limited to lactic acid accumulation, which is caused by long-duration or intense exercise (3, 4). In addition to this muscle fatigue, the sensation of fatigue, which partly resembles mental fatigue in showing lassitude, sleepiness and decreased motivation, often develops after exercise (5-7). Recently, there has been a great increase in the use of over-the-counter supplements and naturally occurring nutraceuticals for the attenuation of physical fatigue (8). 

*Siraitia grosvenorii *(Swingle), a perennial vine of the Cucurbitaceae family, has been cultivated in the restricted area of the southern part of China (9, 10). Fruits of these plants, known as Luo-Han-Guo in China, have been widely used in the treatment of laryngitis, bronchitis, and gastrointestinal diseases in traditional Chinese medicine (11, 12). Studies showed that *Siraitia grosvenorii *fruits extracts (SGFE) possess a wide range of pharmacologic and health-promoting properties including immune enhancement, anti-oxidation, anti-diabetes, anti-tumor and anti-inflammation (13, 14). The main bioactive components of this plant fruits are mogrosides, which are members of the family of triterpene glycosides, including mogroside IVa, mogrosides V and VI, iso-mogroside V, 11-oxomogroside V and siamenoside I (15). Previous studies showed that glycosides are bioactive components of many famous Chinese medicines in recent years and used in developing new drugs (16-19). Triterpene glycosides such as *Siraitia grosvenorii *have been used as an anti-hepatitis drug and steroidal glycosides have been used as anti-epilepsy, anti-inflammatory and anti-tumor glycosides (20-23). However, very little is known about anti-fatigue effects of SGFE. The present study aimed to investigate the effects of SGFE on physical fatigue in mice.

## Experimental

*Chemical*

All chemicals were purchased from Jilin Chemical Reagents Co., Ltd. (Changchun, China) unless otherwise indicated. Commercial diagnostic kits (special for animal testing) used to determine blood lactic acid (BLA) and tissue glycogen were purchased from Nanjing Jiancheng Bioengineering Institute (Nanjing, China). Commercial diagnostic kit used to determine serum urea nitrogen (SUN) was purchased from Biosino Biotechnology Co., Ltd. (Beijing, China). Water was purified with a Milli-Q purification system and was used to prepare all solutions.

*Plant materials*

Fruits of *Siraitia grosvenorii *were purchased from Jilin Pharmaceutical Co. (Changchun, China), and identified by Professor Yang LY, College of Traditional Chinese Medicine, Jilin Agricultural University. Voucher specimens were deposited at the herbarium of Jilin Normal University.

*Preparation of Siraitia grosvenorii fruits extracts*

*Siraitia grosvenorii *fruits extracts (SGFE) were prepared as described previously (24) with slight modifications. Air-dried *Siraitia grosvenorii *fruits (500 g) were ground into powder (particle diameter: 0.2 to 0.5 mm), then the powder was dissolved into 3000 mL distilled water and extracted 3 times at 70°C for 1 h every time. Subsequently, the water-soluble extracts were vacuum concentrated, separated and purified using column chromatography, at last vacuum-dried to yield extracts of *Siraitia grosvenorii *fruits (46.4 g/Kg), which were determined by HPLC system (Waters, Milford, MA). The HPLC system consisted of Waters 1525 binary HPLC pump, Waters 2487 dual λ absorbance detector and a XTerra RP18 column. HPLC analysis indicated that this extracts contained 76.4% glycosides and the major glycoside was mogrosides V (25.27%). Solution of aqueous extract was prepared in saline for the experiment.

*Animals*

Male ICR mice (18-22 g) were purchased from the Jilin Laboratory Animal Breeding and Research Center (Changchun, China). The mice were individually housed in a room maintained at 23 ± 2°C and 50 ± 5% humidity with a 12 h light-dark cycle. They were given free access to food and water throughout the experiments. The experiments were carried out in accordance with the China animal protection law and approved by Ethics Commission of Jilin Normal University.

*Experimental design*

After an adaptation period for a week, the 144 mice were randomly divided into four groups (n = 36 per group): control (C) group, low-dose SGFE-treated (LT) group, middle-dose SGFE-treated (MT) group and high-dose SGFE-treated (HT) group. The animals of control (C) group received an oral administration of physiological saline in a volume of 1.0 mL, and the animals of treated group received the same volume of SGFE (100, 200 and 400 mg/Kg bodyweight/day) for 28 days. The rationale for the selection of the doses was based on our previous experiments and some early literature. The doses of SGFE (100-400 mg/Kg bodyweight) were confirmed to be suitable and effective in the tested mice. The mice were made to swim for 15 min three times a week to accustom them to swimming.

After 28 days, anti-fatigue effects of SGFE were assessed 10 h after the last administration by forced swimming test.

*Forced swimming test*

Twelve mice were taken out from each group to make forced swimming test. The apparatus used in this test was an acrylic plastic pool (90 × 45 × 45 cm) filled with water maintained at 25 ± 2°C. The water in the acrylic plastic pool was 35 cm deep. Mice had a load attached (10% body weight) to its tail to reduce the time of swimming-to-exhaustion tests. Exhaustion was determined by observing loss of coordinated movements and failure to return to the surface within 10 s (25-27) and the swimming time was immediately recorded.

*Analysis of blood biochemical parameters related to fatigue*

Twelve mice were taken out from each group for blood biochemical parameters analyses. Mice were forced to swim for 30 min after weight loading (2% body weight) (25). After resting for 60 min, blood was collected from orbital sinus to examine the blood lactic acid (BLA) and serum urea nitrogen (SUN). Then, BLA and SUN contents were tested according to the recommended procedures provided by the commercial diagnostic kit.

*Analysis of tissue glycogen contents*

Twelve mice were taken out from each group for tissue glycogen contents analyses. Mice were forced to swim for 90 min without loads. After resting for 60 min, the mice were killed to collect their livers and gastrocnemius muscles (28). Then, tissue glycogen contents were tested according to the recommended procedures provided by the commercial diagnostic kit.

*Statistical analysis*

All results are expressed as mean ± SD for ten mice in each group. To determine the effect of treatment, data was analyzed using one-way ANOVA repeated measures. P-values of less than 0.05 were regarded as significant. Significant values were assessed with Duncan’s multiple range test. Data was analyzed using the statistical package “SPSS 12.0 for Windows”.

## Results and Discussion

*Effect of SGFE on body weights of mice*

The body weights of mice were measured at the beginning of the experiment (day 0) and after the administration with different dosages of SGE for 28 days (28^th^ day). It was found (data not shown) that the body weights of mice in the SGFE-treated groups (LT, MT and HT group) were not different significantly from that in the control group (C group) at day 0 or 28 (p > 0.05), which means the SGFE has no effect on body weight. 

*Effect of SGFE on *the time of swimming-to-exhaustion tests *swimming time to exhaustion of mice*

**Figure 1:**
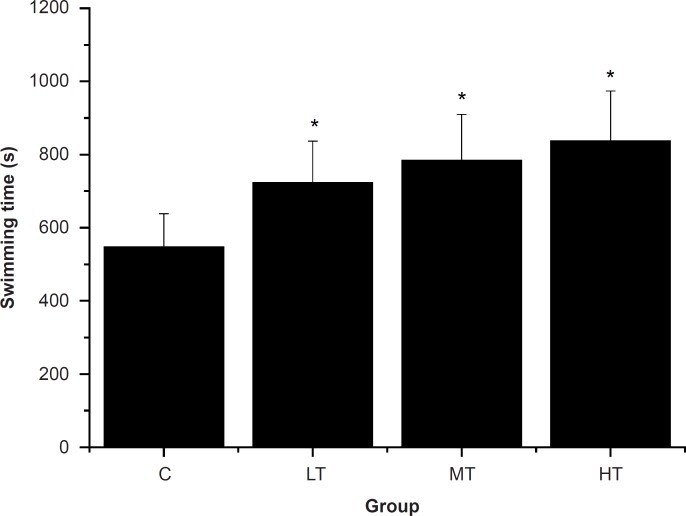
Effect of *Siraitia grosvenorii *fruit extracts (SGFE) on swimming time-to-exhaustion of mice.

A direct measure of an anti-fatigue effect is the increase in exercise tolerance. Swimming-to-exhaustion is an experimental exercise model to evaluate the anti-fatigue effect; it works well for evaluating the endurance capacity of mice and gives a high reproducibility (29). To standardize the workload and reduce the swimming time, weights at specific body weight percentages were added to the chest or tail of the animal (30). Reduced susceptibility to fatigue is correlated with longer swimming times (31). As shown in Figure 1, the swimming time-to-exhaustion of the SGFE-treated groups (LT, MT and HT group) were significantly longer than that of control group (C group) (p < 0.05). The results indicated that SGFE had anti-fatigue effects and these effects were dose-dependent. 

Data were presented as means ± SD (n = 12 per group). C: control, LT: low-dose SGFE-treated (100 mg/Kg bodyweight); MT: middle-dose SGFE-treated (200 mg/Kg bodyweight), HT: high-dose SGFE treated (400 mg/Kg bodyweight). *: p < 0.05 as compared with the control group

*Effect of SGFE on blood lactic acid and serum urea nitrogen of mice*

**Figure 2:**
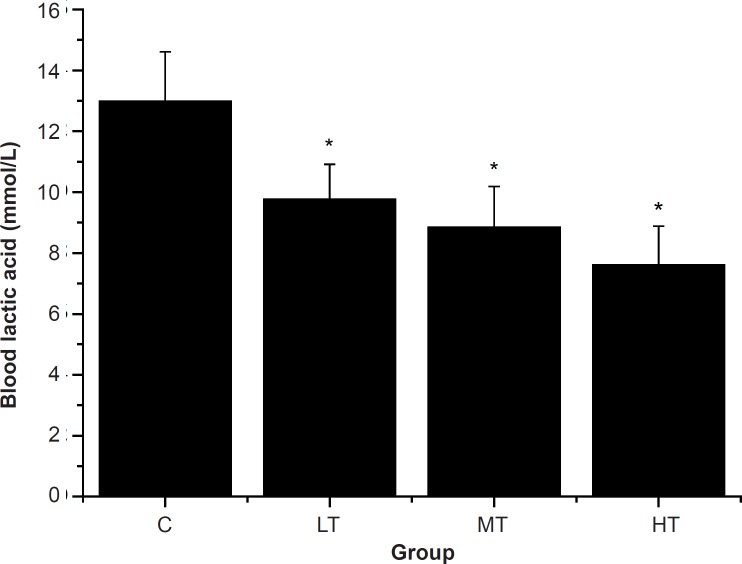
Effect of *Siraitia grosvenorii *fruit extracts (SGFE) on blood lactic acid of mice.

The swimming exercise is known to induce the blood biochemical changes (32). Blood lactic acid (BLA) is the glycolysis product of carbohydrate under an anaerobic condition, and glycolysis is the main energy source for intense exercise in a short time. Therefore, BLA is one of the important indicators for judging the degree of fatigue (28, 33). In other words, BLA represents the degree of fatigue after exercise and the condition of recovery (26). As shown in Figure 2, after swimming, the SGFE-treated groups (LT, MT and HT group) showed a dose-dependent decrease in the BLA levels compared with the control group (C group) (p *< *0.05). The results indicated that SGFE can effectively delay the increase of lactic acid in the blood and postpone the appearance of physical fatigue. 

Data were presented as means ± SD (n = 12 per group). C: control, LT: low-dose SGFE-treated (100 mg/Kg bodyweight); MT: middle-dose SGFE-treated (200 mg/Kg bodyweight), HT: high-dose SGFE-treated (400 mg/Kg bodyweight). *: p < 0.05 as compared with the control group.

**Figure 3:**
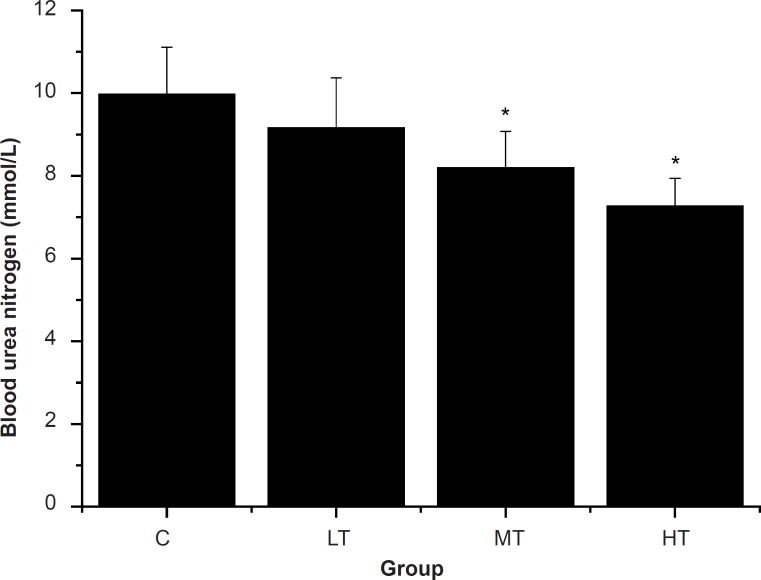
Effect of *Siraitia grosvenorii *fruit extracts (SGFE) on serum urea nitrogen (SUN) of mice.

Blood urea nitrogen (BUN), the metabolism outcome of protein and amino acid, is a sensitive index to evaluate the bearing capability when human bodies suffer from a physical load. Several studies have shown that the BUN in the blood rises significantly for a long-run athlete after exercise (26, 34, 35). In other words, the worse the body is adapted for exercise tolerance, the more significantly the BUN level increases (29, 36). Therefore, BUN is another index of fatigue status. As shown in Figure 3, after **s**wimming, MT and HT group showed a significant decrease in the BUN levels compared with the control group (C group) (p < 0.05). However, BUN levels of the LT group were lower than that of the control group (C group), but there was no significant difference (p > 0.05). The results indicated that SGFE may reduce the catabolic decomposition of protein for energy.

Data were presented as means ± SD (n = 12 per group). C: control, LT: low-dose SGFE treated (100 mg/Kg bodyweight); MT: middle-dose SGFE treated (200 mg/Kg bodyweight), HT: high-dose SGFE treated (400 mg/Kg bodyweight). *: p < 0.05 as compared with control group

*Effect of SGFE on liver and muscle glycogen of mice*

It was known that the endurance capacity of body was markedly decreased if the energy was exhausted (37). Energy for exercise is derived initially from the breakdown of glycogen, after the strenuous exercise muscle glycogen exhausts, and later, energy forms circulating glucose released by the liver (38, 39). Thus, the glycogen contents are sensitive parameters related to fatigue. Table 1 shows the effect of SGFE on liver and muscle glycogen of mice. After swimming, the SGFE-treated groups (LT, MT and HT group) showed a dose-dependent increase in the liver and muscle glycogen contents compared with the control group (C group) (p < 0.05). The results indicated that the rate of glycogen depletion was delayed in the SGFE-treated groups, and suggested that SGFE could decrease carbohydrate utilization during the exercise.

**Table 1:** Effect of *Siraitia grosvenorii *fruits extracts (SGFE) on liver and muscle glycogen of mice.

**Group**	**Liver glycogen (mg/g)**	**Muscle glycogen (mg/g)**
**C**	7.54 ± 2.21	1.21 ± 0.27
**LT**	14.27 ± 3.38*	1.86 ± 0.42*
**MT**	17.29 ± 3.87*	1.95 ± 0.33*
**HT**	19.63 ± 3.11*	2.06 ± 0.61*

Data were presented as means ± SD (n = 12 per group). C: control, LT: low-dose SGFE treated (100 mg/Kg bodyweight); MT: middle-dose SGFE treated (200 mg/Kg bodyweight), HT: high-dose SGFE treated (400 mg/Kg bodyweight). *: p < 0.05 as compared with the control group.

In conclusion, the data showed that SGFE can extend the swimming time of the mice, as well as increasing the liver and muscle glycogen contents, but it decreases the blood lactic acid and serum urea nitrogen levels. These results indicated that SGFE had significant anti-fatigue effects on mice and these effects were dose-dependent. However, further study is needed to elucidate the more exact mechanism of the anti-fatigue effect of SGFE at the cellular and molecular levels.
